# Development and validation of a radiogenomics model to predict axillary lymph node metastasis in breast cancer integrating MRI with transcriptome data: A multicohort study

**DOI:** 10.3389/fonc.2022.1076267

**Published:** 2022-12-29

**Authors:** Huifang Chen, Xiaosong Lan, Tao Yu, Lan Li, Sun Tang, Shuling Liu, Fujie Jiang, Lu Wang, Yao Huang, Ying Cao, Wei Wang, Xiaoxia Wang, Jiuquan Zhang

**Affiliations:** Department of Radiology, Chongqing University Cancer Hospital, Chongqing, China

**Keywords:** radiogenomics, lymph node metastasis, breast cancer, genomics, radiomics

## Abstract

**Introduction:**

To develop and validate a radiogenomics model for predicting axillary lymph node metastasis (ALNM) in breast cancer compared to a genomics and radiomics model.

**Methods:**

This retrospective study integrated transcriptomic data from The Cancer Genome Atlas with matched MRI data from The Cancer Imaging Archive for the same set of 111 patients with breast cancer, which were used as the training and testing groups. Fifteen patients from one hospital were enrolled as the external validation group. Radiomics features were extracted from dynamic contrast-enhanced (DCE)-MRI of breast cancer, and genomics features were derived from differentially expressed gene analysis of transcriptome data. Boruta was used for genomics and radiomics data dimension reduction and feature selection. Logistic regression was applied to develop genomics, radiomics, and radiogenomics models to predict ALNM. The performance of the three models was assessed by receiver operating characteristic curves and compared by the *Delong* test.

**Results:**

The genomics model was established by nine genomics features, and the radiomics model was established by three radiomics features. The two models showed good discrimination performance in predicting ALNM in breast cancer, with areas under the curves (AUCs) of 0.80, 0.67, and 0.52 for the genomics model and 0.72, 0.68, and 0.71 for the radiomics model in the training, testing and external validation groups, respectively. The radiogenomics model integrated with five genomics features and three radiomics features had a better performance, with AUCs of 0.84, 0.75, and 0.82 in the three groups, respectively, which was higher than the AUC of the radiomics model in the training group and the genomics model in the external validation group (both *P* < 0.05).

**Conclusion:**

The radiogenomics model combining radiomics features and genomics features improved the performance to predict ALNM in breast cancer.

## Introduction

Breast cancer is the most commonly diagnosed cancer among women worldwide and is the second leading cause of cancer-related death (1). Axillary lymph nodes (ALNs) are an important path for lymph node metastasis (LNM) in breast cancer. Axillary lymph node metastasis (ALNM) is an important factor affecting the treatment and prognosis of breast cancer patients. Thus, accurate identification of ALN involvement in patients with breast cancer is essential for prognosis and therapeutic decision-making (2). Sentinel lymph node (SLN) biopsy is now considered as the reference standard for ALN status staging in patients with clinically negative lymph nodes (3). Compared with ALN dissection, it significantly reduces complications such as arm numbness, upper extremity edema, nerve damage, etc. (4, 5). However, the procedure is still invasive. Therefore, a noninvasive and reliable assessment of ALN status pretreatment is critical for clinical decision-making.

Magnetic resonance imaging (MRI) is one of the important auxiliary examination methods widely used for breast cancer patients before surgery due to its high sensitivity for evaluating tumor extension, intraductal spread, and the presence of multicentric or multifocal lesions (6). Radiomics based on MRI as a noninvasive technology has emerged as a potential method in precision medicine for breast cancer (7, 8). Recently, radiomics has been widely used to predict LNM in breast cancer and has demonstrated excellent predictive performance (9–11). Radiomics focuses on the systematic characterization of the aggressiveness of breast cancer by effectively extracting and analyzing massive image data. However, radiomics may lack the ability of tumor characterization in microstructural features.

Previous efforts have been made to explore the identified genomics features, which provide a powerful tool for identifying breast cancer patients with distant recurrence and might provide a better method for individual risk assessment in patients with lymph node-negative breast cancer (12). Other studies revealed that a miRNA-dependent model could predict LNM in cervical cancer patients (13), and an epigenetic model could predict axillary staging with ER-positive early-stage breast cancer patients (14). The greatest limitation to deploying genome sequencing for clinical application is that tumor spatial heterogeneity limits genomics tissues, and genomics only reflect a microcosm of the genetic code.

Radiogenomics is a rapidly developing method to integrate genomics data with radiomics data (15). Radiogenomics can provide voxel-by-voxel information from genomics to tumor imaging and thereby guide tailored therapy and help to improve our understanding of tumor biology (15). In the earliest radiogenomics study of breast cancer, Yamamoto et al. explored the relationship between MRI features and gene expression (16). A follow-up study that included preoperative DCE-MRI explored the multiscale relationships between DCE-MRI phenotypes, metastasis, and long noncoding RNA expression (17). These results mainly explored the relationships between imaging and genomics. Furthermore, studies have reported that radiogenomics models have a higher prediction performance than genomics-only models in predicting LNM or radiomics-only models in predicting pathologic complete response in triple-negative breast cancer (18, 19). One possible reason is that radiogenomics can simultaneously provide information regarding macroscopic and microscopic features of the tumor tissue.

Hence, the purpose of this study was to develop and validate a radiogenomics model for the prediction of ALNM in breast cancer compared to a genomics model and a radiomics model.

## Materials and methods

### Participant characteristics

**Figure 1:**
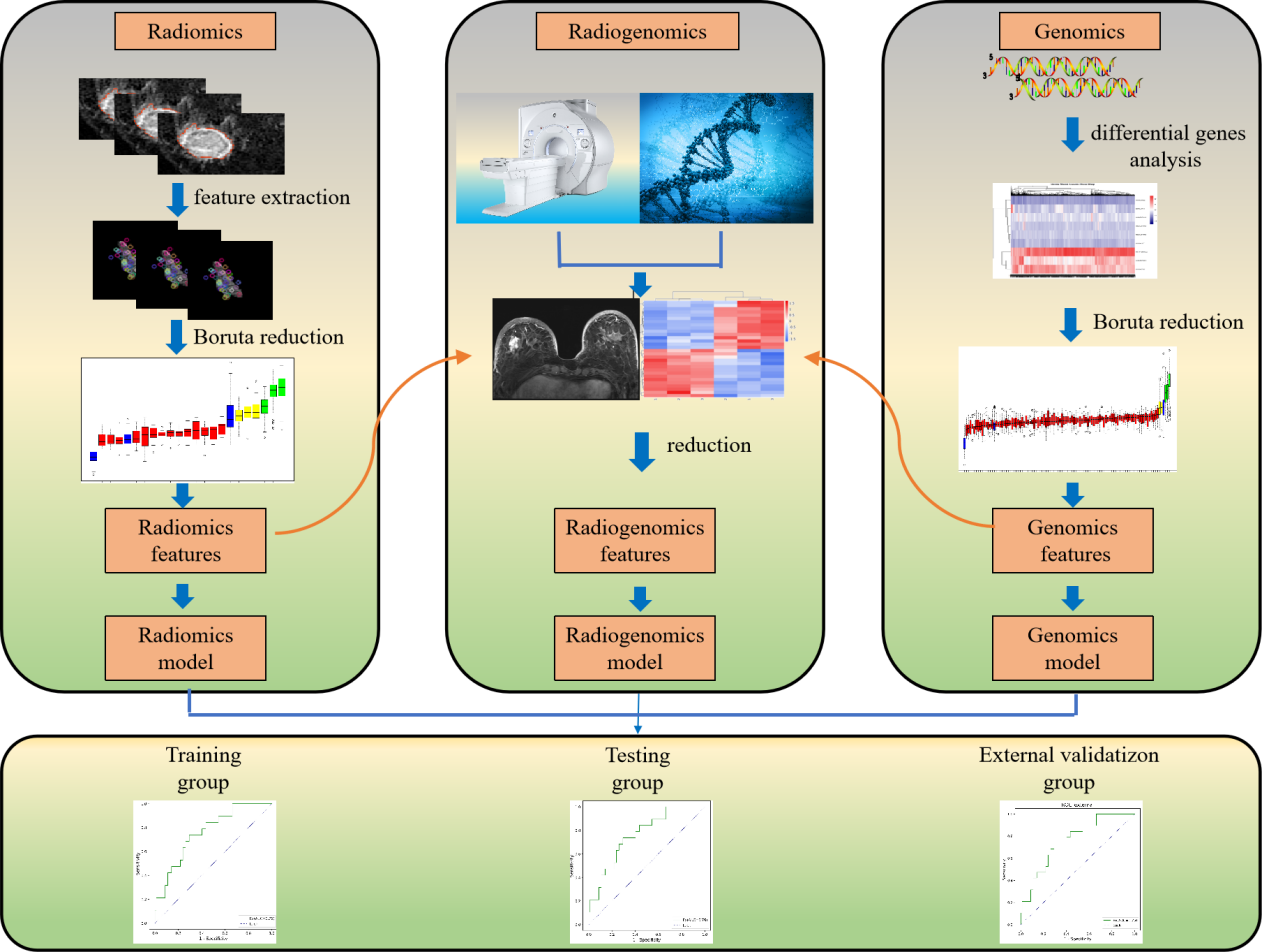
Framework overview.

This study was conducted with patients retrospectively enrolled from a public database and our hospital. The public database was originally submitted to The Cancer Genome Atlas (TCGA) and the Cancer Imaging Archive (TCIA) by the contributing institutions under an Institutional Review Board-approved protocol. The second part of the study was approved by the ethics committee of our hospital, and the requirement for individual consent for this retrospective analysis was waived between January 2021 and December 2021. Clinical, breast MRI, and transcriptome data for all patients from TCGA and TCIA, and our hospital were integrated and analyzed. A general overview of the analysis protocol is shown in **Figure 1**.

**Figure 2:**
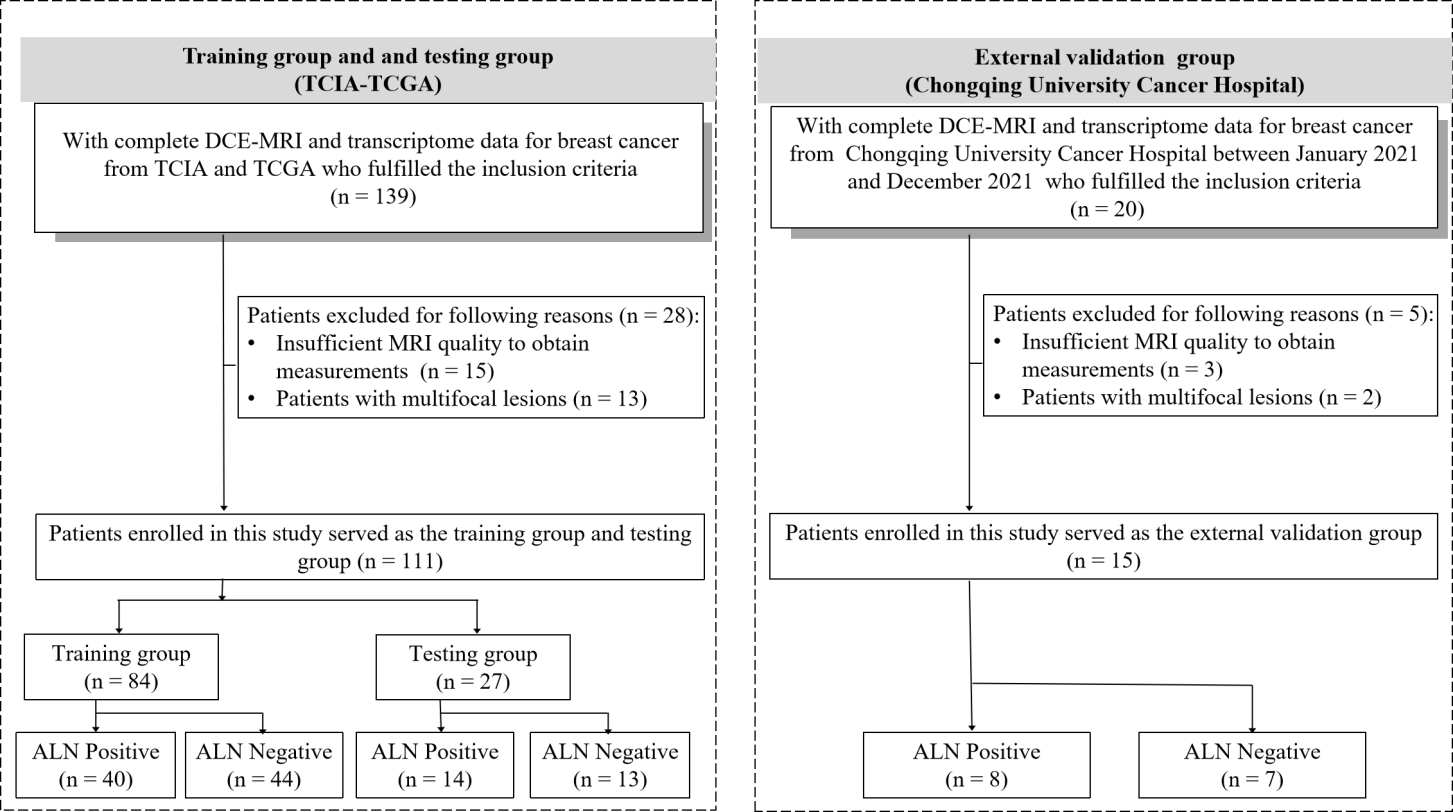
Patient recruitment workflow. DCE, dynamic contrast enhanced; TCGA, The Cancer Genome Atlas; TCIA, The Cancer Imaging Archive; ALN, axillary lymph node.

The inclusion criteria were as follows: (i) patients had histologically confirmed unilateral primary breast cancer; (ii) patients who underwent breast surgery without neoadjuvant chemotherapy, and sentinel lymph node biopsy or ALN dissection with curative intent; (iii) patients with suspected positive ALN by clinical and/or imaging examinations and plan to receive neoadjuvant therapy, lymph node status was determined by ultrasound-guided core needle biopsy before neoadjuvant therapy (20, 21); (iv) availability of clinical data (age), T staging, complete pathological data for ALN and molecular subtype; (v) breast DCE-MRI was conducted before anti-tumor treatment and core needle biopsy for evaluating tumor extension, intraductal spread, and the presence of multicentric or multifocal lesions; and (vi) availability of transcriptome data. The exclusion criteria were as follows: (i) insufficient MRI quality to obtain measurements and (ii) patients with multifocal lesions. (iii) patients with negative ALN by clinical and/or imaging examinations and plan to receive neoadjuvant therapy. Details are provided in **Figure 2**.

### Genomics data analysis

Fifteen patients with breast cancer from our hospital were used for the external validation group. Postoperative tumor samples were extracted from the primary tumor sites, and snap-frozen breast cancer samples were collected by the Tumor Samples and Genetic Information Resource Library. Next, the sample from the hospital was processed using RNA extraction and sequencing. Total RNA was extracted from tissue samples with TRIzol reagent (Invitrogen, Carlsbad, CA, USA). RNA purity was monitored on 1% agarose gel. Then, cDNA was synthesized from total RNA using the PrimeScript RT reagent Kit with gDNA Eraser (TaKaRa, RR047A). Then, all patients’ transcriptomic data were analyzed for differentially expressed genes (DEGs). The DEGs between the groups with and without ALNM were screened using DESeq (http://bioconductor.org/packages/release/bioc/html/DESeq.html) to detect DEGs with gene symbol annotation with thresholds of *P* < 0.05 and |fold change| > 2.

### DCE-MRI data acquisition

The public MRI data of breast cancer were downloaded from TCIA. The breast cancer MRI was performed at our hospital using a commercially available 1.5-T system (Philips Ingenia, the Netherlands) with an eight-channel breast array coil. Routine precontrast MRI included T1-weighted imaging, fat-suppressed T2-weighted imaging, and diffusion-weighted imaging. The gadolinium contrast agent (Hengrui, Jiangsu, China) was injected at a rate of 2 mL/s for a dose of 0.1 mmol/kg, followed by a 20 mL saline flush at a rate of 2 mL/s. One precontrast phase image and seven postcontrast phase images were acquired using the axial e-THRIVE polyphase sequence with the following parameters: TR/TE = 5.1/2.5 ms; matrix = 252 × 348; flip angle = 10°; pixel size = 1 × 1 mm; and slice thickness = 2 mm.

### Radiomics feature extraction

The regions of interest (ROIs) were delineated manually in each slice of the T1+C data (the peak-enhanced phase of the multiphase contrast-enhanced MRI selected according to the time-intensity curve) by excluding the air, necrosis, and calcification areas *via* the Dr. Wise Multimodal Research Platform (https://keyan.deepwise.com,V1.6.3) (Beijing Deepwise & League of PHD Technology Co., Ltd., Beijing, China). Each DCE-MRI case was reviewed by two experienced radiologists (XXW and LL with 9 and 8 years of experience, respectively). After manual segmentation, consistency intraclass correlation coefficient (ICC) (22) analysis was performed to measure the features’ observer repeatability. ICC scores greater than 0.8 are generally considered to indicate good repeatability. We standardized the image processing procedure according to the image biomarker standardization initiative reference manual (23). Next, 1651 radiomics features were extracted from the normalized image using Pyradiomics (http://www.radiomics.io/pyradiomics.html). Then, they were normalized with Z scores to obtain a standard normal distribution of image intensities. The radiomics features were composed of the following eight types of features: first-order statistics, shape-based (3D), shape-based (2D), gray level cooccurrence matrix (GLCM), gray level run length matrix (GLRLM), gray level size zone matrix (GLSZM), neighboring gray-tone difference matrix (NGTDM), gray level dependence matrix (GLDM). All of these features have generally been used in previous radiomics studies (24, 25).

### Genomics and radiomics feature selection

We used the Boruta method (R3.6.1 with Boruta version 5.2.0) from a coarse to fine feature reduction strategy to reduce both genomics and radiomics features (24). It was divided into the two steps described below. (i) Univariate analysis was performed using *Student’s t* test or the *Mann–Whitney U test* to compare genomics and radiomics features between the groups with and without LNM. All features were ranked in ascending order according to the *P* value, and the top 5% of features were retained for further analysis. (ii) *Spearman* correlation analysis was used to eliminate redundant features. All genomics and radiomics features with correlation coefficients > 0.85 were detected, and the features with lower *P* values were retained. Then, the selected features were used for further model construction and validation.

### Model construction and validation

Patients from the TCIA-TCGA were randomly divided into training and testing group at a ratio of 3:1, and 15 patients from our hospital were enrolled as the external validation group. Logistic regression analysis (Python3.7 with sklearn version 1.1.2) was used to construct radiomics, genomics and radiogenomics models for predicting ALN status. The radiogenomics model was conducted through dimensionality reduction by integrating the features of the radiomics model and the features of the genomics model. All models were trained based on TCGA datasets, and fivefold cross validation was used to determine the parameters of the logistic regression models. We evaluated the prediction performance of the radiomics, genomics and radiogenomics models in the training, testing and external validation groups by constructing receiver operating characteristic (ROC) curves and calculating areas under the curves (AUCs). Decision curve analysis was applied to estimate the clinical utility of the three models. In addition, a calibration curve was generated to evaluate the consistency between the predicted value and the true value.

### Statistical analysis

Statistical analyses were performed by commercially available statistical software (SPSS software, version 25.0; Armonk, US). Descriptive statistics were summarized as the means ± standard deviations. Categorical variables were expressed as numbers. Continuous clinical variables were compared using *Student’s t* test or the *Mann–Whitney U test*. For categorical variables, *chi-square* tests were used to test differences between groups. The performances of the radiomics model, genomics model and radiogenomics model were compared using the *Delong* test. For all tests, *P* < 0.05 was considered as statistically significant.

## Results

### Baseline characteristics

The baseline characteristics in the training, testing, and external validation group are presented in **Table 1**. A total of 126 patients with breast cancer from one hospital and public database were enrolled in the study. The training group (53.39 ± 11.44 years) included 84 patients, and the testing group (55.96 ± 10.56 years) included 27 patients from the TCGA and TCIA datasets. The external validation group (50.53 ± 7.24 years) included 15 patients who were from our hospital. There were no significant differences in age or LNM among the three groups (*P* = 0.91 and 0.92).

**Table 1:** The clinical characteristics of patients in the training, testing and external validation groups.

Characteristics	Training group (n=84)	Testing group (n=27)	External validation group (n=15)	*P* value
Age, mean ± SD, years (range)	53.39 ± 11.44 years (range, 29~80 years)	55.96± 10.56 years (range, 31~82 years)	50.53 ± 7.24 years (range, 42~65years)	0.91
Clinical stage				0.00
I	16	7	3	
II	57	17	7	
III	11	3	5	
T stage				0.00
T1	33	12	4	
T2	45	14	10	
T3	6	1	1	
Axillary lymph node status				0.92
Positive	40	13	8	
Negative	44	14	7	
Molecular subtype				0.00
Luminal A	52	20	3	
Luminal B	11	3	8	
HER2-enriched	6	1	4	
Basel-like	15	3	0	
	

SD, standard deviation; HER2, human epidermal growth factor receptor 2.

### Development and validation of the genomics model

**Figure 3:**
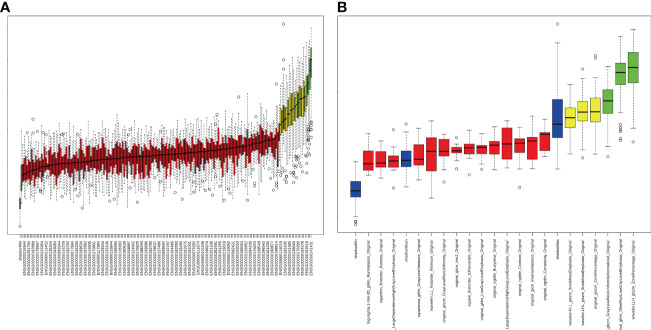
Strategy for feature selection using the Boruta method. **(A)** Genomics feature selection; **(B)** Radiomics feature selection.

**Figure 4:**
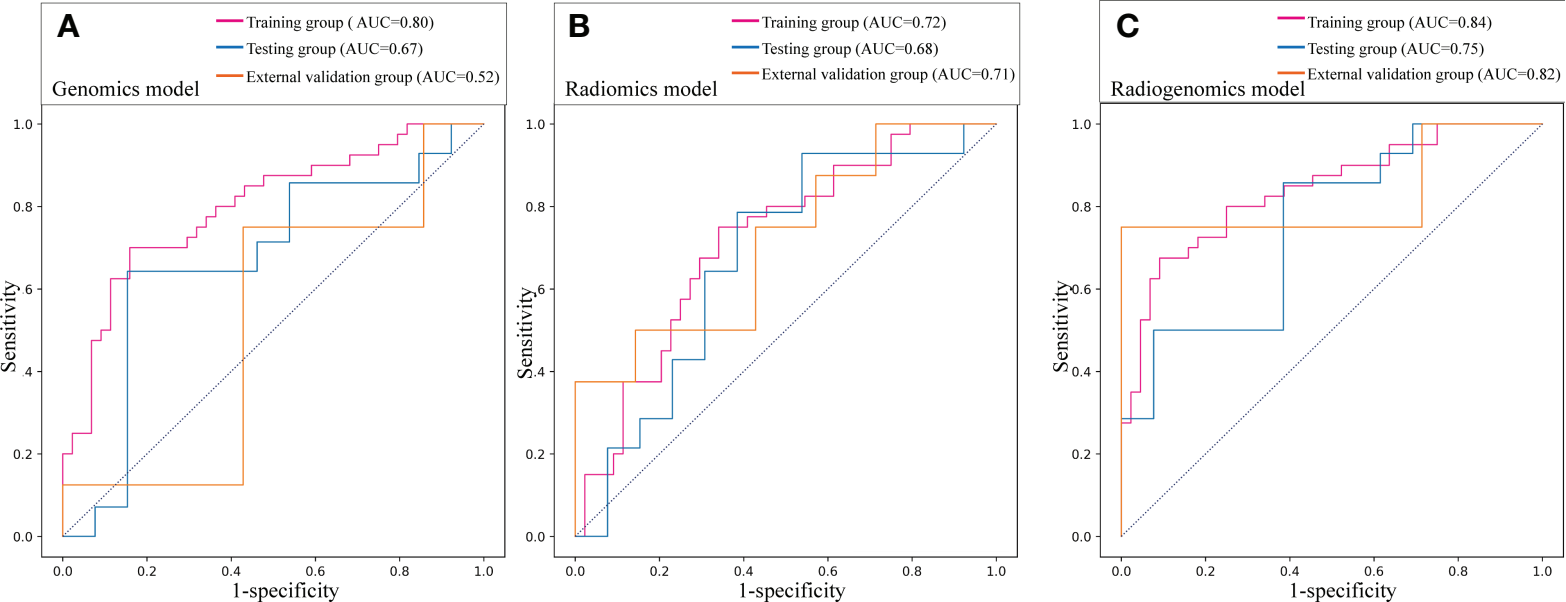
Prediction performance of the three models in the training, testing and external validation groups. **(A)** Receiver operating characteristic (ROC) curve of the genomics model; **(B)** ROC curve of the radiomics model; **(C)** ROC curve of the radiogenomics model.

For differential gene expression analysis, 136 DEGs were found between patients with and without ALNM (**Figure S1**). As shown in **Figure S1A**, expression levels of 94 genes were increased and 42 genes were decreased between patients with and without ALNM. Heatmap representation of DEGs revealed a similarity between patients with and without ALNM (**Figure S1B**). Then, 9 genomics features were selected through Boruta analysis (**Figure 3A**). The 9 genomics features were used to develop the genomics model for predicting ALNM in breast cancer. The AUCs of the training, testing, and external validation group were 0.80, 0.67 and 0.52, respectively (**Figure 4A**). The AUC and corresponding sensitivity, specificity, positive predictive value (PPV), and negative predictive value (NPV) values of the genomics model are displayed in detail in **Table 2**. The calibration curves for the probability of the genomics model for ALNM in the training group, testing group and external validation group are shown in **Figures S2A–C**.

**Table 2:** The performance of three models in the training, testing and external validation groups.

Characteristics	Training group	Testing group	External validation group
Genomics model			
AUC	0.80	0.67	0.52
Sensitivity (%)	70.00	71.43	37.50
Specificity (%)	84.09	46.15	57.14
Accuracy (%)	77.38	59.26	46.67
PPV (%)	80.00	58.83	50.00
NPV (%)	75.51	60.00	44.44
Radiomics model			
AUC	0.72	0.68	0.71
Sensitivity	75.00	78.57	50.00
Specificity	65.91	53.85	57.14
Accuracy	70.24	66.67	53.33
PPV (%)	66.67	64.71	57.14
NPV (%)	74.36	70.00	50.00
Radiogenomics model			
AUC	0.84	0.75	0.82
Sensitivity	67.50	71.43	62.50
Specificity	90.91	61.54	100.00
Accuracy	79.76	66.67	80.00
PPV (%)	87.10	66.67	100.00
NPV (%)	75.47	66.67	70.00


PPV, positive predictive value; NPV, negative predictive value.

### Development and validation of the radiomics model

A total of 1651 features were extracted from DCE-MRI from every patient. After the reduction by Boruta (**Figure 3B**), 3 radiomics features were used to construct and validate the radiomics model. The AUCs of the prediction performance of ALNM in breast cancer of the radiomics in the training and testing group were 0.72 and 0.68, respectively (**Figure 4B**). In addition, the performance of the radiomics model was validated in an independent external validation group, and the AUC of the validation group was 0.71. The AUC and corresponding sensitivity, specificity, PPV and NPV values of the radiomics model are displayed in detail in **Table 2**. The calibration curves for the probability of the radiomics model for ALNM in the training group, testing group and external validation group are shown in **Figures S2D–F.**

### Development and validation of the radiogenomics model

**Figure 5:**
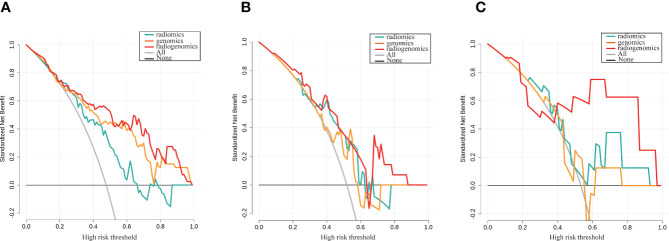
Decision curve analysis for the genomics, radiomics and radiogenomics models of LNM prediction in breast cancer. **(A)** Training group; **(B)** Testing group; **(C)** External validation group. The y-axis measures the standardized net benefit.

After stepwise logistic regression with both direction, 5 genomics features and 3 radiomics features were finally selected to develop a radiogenomics model. We performed correlation analysis between the 3 radiomics and 5 genomics features, as shown in **Table S1**. The results showed that the correlations between the genomics and radiomics features were slightly weaker. The AUCs of predicting ALNM for the radiogenomics model in the training group, testing group and external validation group were 0.84, 0.75 and 0.82, respectively (**Figure 4C**). The AUC and corresponding sensitivity, specificity, accuracy, PPV, and NPV values of the three groups are detailed in **Table 2**. Good agreement of the radiogenomics model between the observation and prediction was assessed by the calibration curve, which showed that the bias-corrected line lay close to the ideal curve in the training, testing, and validation group (**Figures S2G–I**). In addition, the decision curve showed that the radiogenomics model could add more benefit to the prediction of ALNM than the genomics model and radiomics model in the three groups (**Figure 5**).

**Figure 6:**
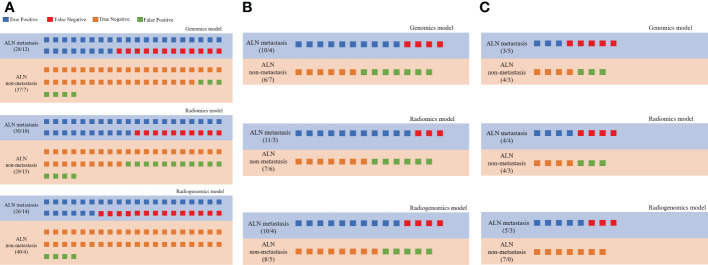
Prediction profiles of the three models. All individual participants were identified as “LNM” or “non-LNM”, and recognized as true positive (blue), false negative (red), true negative (orange) and false positive (green) according to their true labels of LNM in all groups. **(A)** Prediction profiles of the training group; **(B)** Prediction profiles of the testing group; **(C)** Prediction profiles of the external validation groups.

The prediction profiles of the radiogenomics model compared with the genomics model and radiomics model by the *DeLong* test in the training group, testing group and external validation group are shown in **Figure 6**. The results showed that the radiogenomics model significantly improved the AUC compared with the radiomics model in the training group (*P* = 0.01) and showed superior performance compared with the genomics model in the external validation group (*P* = 0.02) (**Table 3**).

**Table 3:** Delong test for prediction improvements of radiogenomics model in the training, testing and external validation groups.

Characteristics	Training group	*P* value	Testing group	*P* value	External validation group	*P* value
Radiomics model vs. Genomics model						
*DeLong* test	-0.08	0.27	0.01	0.97	0.19	0.41
radiogenomics model vs. Radiomics model						
*DeLong* test	0.12	**0.01**	0.07	0.43	0.11	0.50
radiogenomics model vs. Genomics model						
*DeLong* test	0.04	0.28	0.08	0.35	0.30	**0.02**

The bold values represent a p-value less than 0.05, indicating a significant improvement in prediction.

## Discussion

In this study, we established three models for the assessment of ALNM in patients with breast cancer. The radiogenomics model incorporating quantitative radiomics and genomics features could accurately predict LNM with favorable AUC, high specificity, and PPV, which were superior to those of the radiomics and genomics model. The robustness and generalizability of the radiogenomics model were further tested in a multicohort and validated in one hospital.

Preoperative LNM prediction could be beneficial for breast cancer patients. Previous studies have identified that genomics and epigenomic markers unraveled significant epigenetic changes during the progression from primary breast tumor to LNM, which may contribute to improved prognosis and prediction in breast cancer (26). The gene expression data could predict LNM, and the value of such patterns resulted in a predictive accuracy of approximately 90% (27). In this study, we developed a genomics model consisting of 9 features with good performance in predicting LNM. These genes included PTPN21 (28), ST6GALNAC3 (29), FAM13A (30), and CHRNA7 (31), which were correlated with metastasis. These genes were also enriched in the PI3K/Akt, Notch1/Hes1 or Akt1/mTOR signaling pathways and were reported to be correlated with LNM in breast cancer (32–35). The NMRK2 gene was identified as an important target for mitochondrial respiration, and mitochondrial respiration is frequently dependent on metastatic cells (36, 37). A study showed that the recurrence-free survival of patients with loss of function of ZFP36L2 was significantly shorter than that of patients with no loss of ZFP36L2 function in colorectal cancer (38).

The results of our study confirmed that the radiomics model could noninvasively predict ALNM in breast cancer. Previous research has shown that the radiomics model alone predicted LNM with AUCs of 0.76 (39) and 0.806 (40) in breast cancer patients, and their prediction ability was moderate. When the above radiomics features were combined with clinical features or clinicopathologic characteristics, the AUC improved significantly. However, our present study enrolled patients from TCIA, which is a multiagency mixed data, and the radiomics features were not combined with clinical features; therefore, the AUC values of the radiomics model were lower than those of previous studies.

Radiogenomics is an emerging field of cancer research. The first study on radiogenomics in breast cancer was published in 2012 (16), which revealed that radiogenomics analysis of breast cancer with MRI is a novel method that can be used to understand the underlying molecular biology of breast cancers. Then, an increasing number of studies have mainly focused on exploring the correlation between the morphological and enhancement features of DCE-MRI and the genomics features of molecular subtypes (41, 42). In our results, the radiogenomics model had a higher predictive performance than the radiomics-only model or the genomics-only model to predict ALNM in breast cancer because it incorporated macroscopic genomics features and high-throughput radiological features to enhance the predictive value and discover novel biomarkers. Our results showed that genomics features have few associations with MRI-derived radiomics features, which suggests that genes provide additional information for structural imaging. Radiogenomics can effectively predict LNM by bridging the limitations of genomics and radiomics and assisting clinicians in making more precise clinical decisions.

The 2022 NCCN Clinical Practice Guidelines in Oncology recommend that ultrasound-guided fine-needle aspiration cytology or core needle biopsy can be performed for patients with lymph nodes suspected to be positive by clinical and/or imaging examinations or patients considering systemic therapy before surgery (43). Previous study (44) confirmed that ultrasound-guided core needle biopsy was superior to ultrasound-guided fine-needle aspiration cytology in diagnosing axillary nodal metastases: sensitivity 88% (95 confidence interval: 84% to 91%) versus 74% (95 confidence interval: 70% to 78%) respectively, and they both a high specificity of 100%. In our study, we used US guided core needle biopsy in diagnosing ALN metastasis in patients who received neoadjuvant chemotherapy. Therefore, this ensures the reliability and accuracy of this study.

Our study had some limitations. First, this was a retrospective study, and a larger sample size would be desirable. Given the lack of multicenter data, we compiled the training and testing groups from the TCIA-TCGA and a verification group from our hospital database, thereby extending the generalizability of the model. Second, we only focused our prediction on DCE without considering other MRI techniques, such as T2-weighted imaging and DWI. Finally, only logistic regression analysis was used to construct the prediction model, and future studies should consider deep learning or more machine learning algorithms.

By integrating radiomics and genomics features, we built a radiogenomics prediction model that can significantly improve the performance to predict ALNM in breast cancer. The radiogenomics prediction model might reduce unnecessary ALN dissection and improve the quality of life of cancer patients, which could contribute to the realization of precision medicine in breast cancer.

## Data availability statement

The data presented in the study are deposited in the European Nucleotide Archive repository, accession number ERR10628595, ERR10628596, ERR10628597 https://www.ebi.ac.uk/ena/browser/.

## Ethics statement

The studies involving human participants were reviewed and approved by The public database was originally submitted to The Cancer Genome Atlas (TCGA) and the Cancer Imaging Archive (TCIA) by the contributing institutions under an Institutional Review Board-approved protocol. The second part of the study was approved by the ethics committee of our hospital(CZLS2022030-A). Written informed consent for participation was not required for this study in accordance with the national legislation and the institutional requirements.

## Author contributions

Conceptualization, HC and XL. Methodology, XL, FJ and LW. Validation, TY and LL. Data Analysis, HC, XL, SL and ST. Writing – Original Draft Preparation, HC. Writing – Review & Editing, XW and JZ. Supervision, YH, YC, WW. Funding Acquisition, HC, XW and JZ. All authors contributed to the article and approved the submitted version.
