# Supplementary material for: Development and validation of a radiogenomics model to predict axillary lymph node metastasis in breast cancer integrating MRI with transcriptome data: A multicohort study
Source: Front Oncol. 2022 Dec 29;12:1076267. doi: 10.3389/fonc.2022.1076267 (PMC9837803; doi:10.3389/fonc.2022.1076267)
Supplement: Supplementary file 1 [file DataSheet_1.docx]

**Supplementary Materials**

**Figure S1** Overview of the differentially expressed genes of breast cancer patients with and without ALNM. (A) Volcano plots of differentially expressed genes between patients with and without ALNM. (B) Hierarchical clustering of differentially expressed genes between patients with and without ALNM based on log 2-transformed expression values (RPKM).


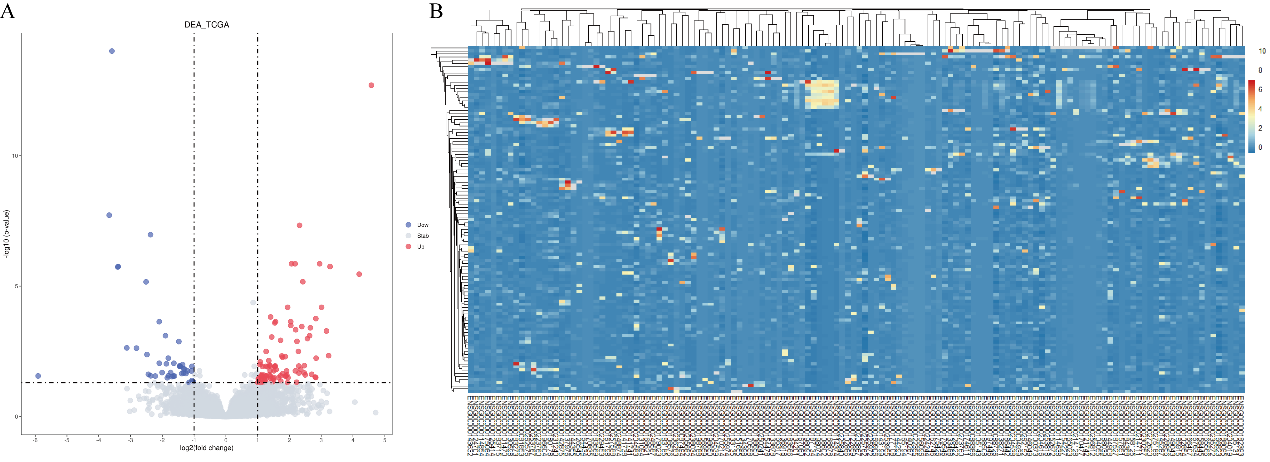


**Figure S2** Calibration curves of the genomics, radiomics and radiogenomics models of ALNM prediction in each group. (A-C) Calibration curve of the genomics model; (D-F) Calibration curve of the radiomics model; (G-I) Calibration curve of the radiogenomics model.


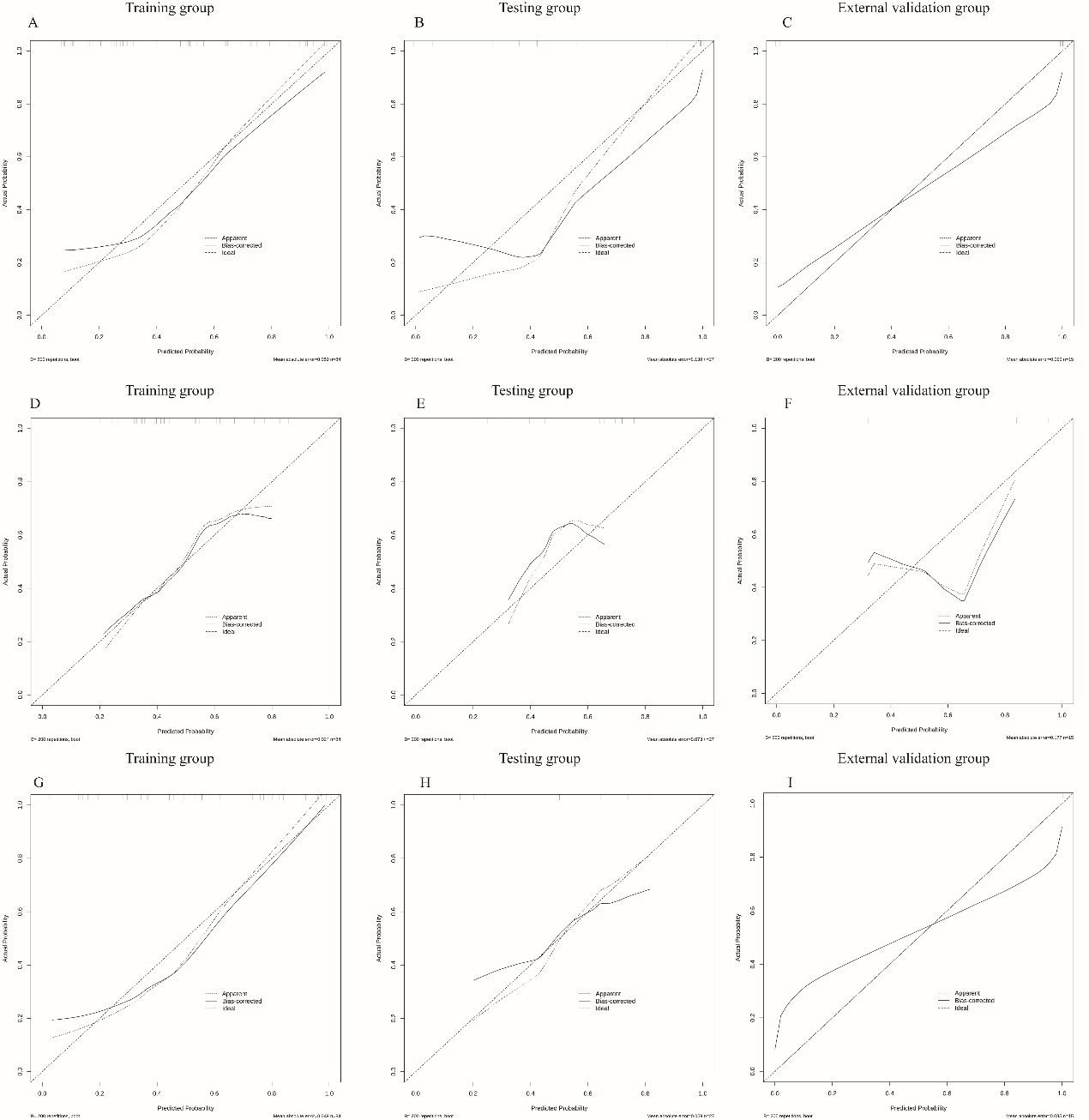


| **Table S1. The correlation coefficient of radiomics and genomics features for prediction ALNM in three groups** | | | | | | | |
| --- | --- | --- | --- | --- | --- | --- | --- |
| Radiomics features | Genomics features | Training group | | Testing group | | External validation group | |
|  |  | *r* value | adjusted *P* value | *r* value | adjusted *P* value | *r* value | adjusted *P* value |
| original_glrlm_ShortRunLowGrayLevelEmphasis_Original | FAM13A | 0.04 | 0.71 | -0.33 | 0.09 | 0.17 | 0.55 |
| wavelet-HHL_glszm_GrayLevelNonUniformityNormalized_Original | FAM13A | -0.07 | 0.54 | 0.18 | 0.36 | 0.01 | 0.97 |
| wavelet-LLH_glszm_ZonePercentage_Original | FAM13A | -0.07 | 0.51 | -0.14 | 0.48 | -0.08 | 0.77 |
| original_glrlm_ShortRunLowGrayLevelEmphasis_Original | ZFP36L2 | 0.00 | 0.97 | -0.27 | 0.17 | -0.05 | 0.87 |
| wavelet-HHL_glszm_GrayLevelNonUniformityNormalized_Original | ZFP36L2 | 0.06 | 0.57 | 0.25 | 0.21 | 0.21 | 0.45 |
| wavelet-LLH_glszm_ZonePercentage_Original | ZFP36L2 | 0.00 | 0.99 | -0.11 | 0.60 | 0.24 | 0.38 |
| original_glrlm_ShortRunLowGrayLevelEmphasis_Original | ASXL3 | 0.18 | 0.10 | -0.34 | 0.08 | 0.11 | 0.69 |
| wavelet-HHL_glszm_GrayLevelNonUniformityNormalized_Original | ASXL3 | 0.06 | 0.61 | 0.13 | 0.53 | 0.01 | 0.98 |
| wavelet-LLH_glszm_ZonePercentage_Original | ASXL3 | 0.06 | 0.59 | 0.38 | 0.05 | -0.19 | 0.51 |
| original_glrlm_ShortRunLowGrayLevelEmphasis_Original | ST6GALNAC3 | 0.14 | 0.20 | -0.33 | 0.10 | 0.25 | 0.37 |
| wavelet-HHL_glszm_GrayLevelNonUniformityNormalized_Original | ST6GALNAC3 | 0.00 | 0.97 | 0.04 | 0.86 | -0.16 | 0.56 |
| wavelet-LLH_glszm_ZonePercentage_Original | ST6GALNAC3 | -0.01 | 0.92 | 0.31 | 0.12 | -0.20 | 0.47 |
| original_glrlm_ShortRunLowGrayLevelEmphasis_Original | PTPN21 | **0.25** | **0.02** | -0.29 | 0.15 | 0.31 | 0.25 |
| wavelet-HHL_glszm_GrayLevelNonUniformityNormalized_Original | PTPN21 | -0.05 | 0.65 | 0.32 | 0.10 | -0.43 | 0.11 |
| wavelet-LLH_glszm_ZonePercentage_Original | PTPN21 | 0.06 | 0.62 | 0.07 | 0.73 | 0.09 | 0.75 |
